# Spatial Language of Young Children During Block Play in Kindergartens in Urban China

**DOI:** 10.3389/fpsyg.2021.568638

**Published:** 2021-02-23

**Authors:** Xiaoli Yang, Yuejuan Pan

**Affiliations:** ^1^Faculty of Education and Sciences, Sichuan Normal University, Chengdu, China; ^2^Faculty of Education, Beijing Normal University, Beijing, China

**Keywords:** block play, spatial language, use, representation, features

## Abstract

Spatial language is an important predictor of spatial skills and might be inspired by peer interaction and goal-oriented building behaviors during block play. The present study investigated the frequency, type and level of children’s spatial language during block play and their associations with the level of block play by observing 228 young children in classrooms equipped with unit blocks and allowing free play on a daily basis. The findings showed that during block play, young children used more words about spatial locations, deictic terms, dimensions, and shapes and fewer words about spatial features or properties and spatial orientations or transformations. Spatial locations were used most frequently, and young children tended to use vertical location words to represent the corresponding location. Most young children used gestures in conjunction with spatial deictic terms. Among shape words, tetragon words were frequently used, and the representation of spatial shapes showed alternatives, collective tendencies and gender differences. The use of spatial language during the play process had a significant positive correlation with age, the construction structure, and form of block building.

## Introduction

Spatial skills in the early years may predict young children’s later academic performance in mathematics, science, engineering, and technology learning (Wai et al., 2009; Newcombe and Frick, 2010; Vasilyeva and Lourenco, 2010; Zhu, 2017) and are an important domain of children’s mathematics learning and development (Wai et al., 2009; Verdine et al., 2014; Lauer and Lourenco, 2016; Verdine et al., 2017; Simoncini et al., 2020). Spatial language is the language used to communicate spatial information to others and represent the location and spatial relationship of objects (Pang et al., 2008). It is also the internal process of thinking, reasoning, and operation of spatial information, which is one of the important forms of children’s external spatial representation (Pang et al., 2008). The use of spatial language enables children to pay attention to and process spatial information (Shusterman and Spelke, 2005), so it may improve the effect of spatial reasoning (Levinson, 2001) and promote the development of spatial skills. Variations in spatial skills can be predicted by differences in children’s use of spatial language (Hermer-Vazquez et al., 2001; Pruden et al., 2011).

Block play is a kind of construction play that combines small blocks into larger objects in a certain way to represent the physical world (Yang et al., 2020). Peer interactions, goal-oriented construction behaviors and the related thematic context in block play can inspire children’s spatial language (Casey et al., 2008; Ferrara et al., 2011). Many studies have focused on the family environment and children’s spatial language, but few studies have analyzed the association between children’s construction level and their use of spatial language in block play.

To support young children’s spatial skills in kindergarten classrooms, it is necessary to investigate the frequency, type and level of young children’s spatial language in the context of block play and their association with the level of block play.

### Children’s Spatial Language

Spatial language is the representation of spatial relations. Constructing and understanding the relationship between spatial cognition and the symbolic system is key to the development of spatial skills (Ferrara et al., 2011). Spatial language provides children with a representative system of spatial concepts to identify and code spatial clues (Miller et al., 2016) and understand spatial categories. Mastery of spatial language supports children’s understanding of spatial concepts, provides children with classification experience (Bowerman and Choi, 2003, p. 387–428), and guides children to pay attention to the spatial environment (Ferrara et al., 2011). Moreover, children can recall relevant spatial information by describing the spatial properties of objects and events (Loewenstein and Gentner, 2005). Zhang et al. (2011) tested non-blind children, congenitally blind children, and acquired blind children. They found that visual loss blind children determined the features of organizing spatial concepts, and that language played an important role in this process. Spatial language can influence how people represent and reason about space (Hermer-Vazquez et al., 2001; Loewenstein and Gentner, 2005).

Many researchers classify spatial language according to its contents. The spatial language system in linguistics is divided into two sections. One is external spatial language, such as spatial relations (on the table), landmarks (come to me), and observers (in his left). The other is internal spatial language, in general, including spatial shapes (strip and bulk) and spatial metric terms (square meter and step), partially including the edge of space with objects at the center (corner) and parts of the human body (face, nose, and head) (Zhao, 2008, p. 82–90). An and Wu (2019) divided spatial language into two dimensions: spatial locations and spatial tendency words. Studies by Ferrara et al. (2011) and Levine et al. (2012) are more specific and detailed. Based on previous literature, this study classified children’s spatial language into spatial locations (up and down), deictic terms (here and there), dimensions (long and tall), spatial features or properties (curvy and straight), shapes (rectangle and square), and spatial orientations or transformations (“turn it around,” “the man is facing the block”).

Chinese children show specific features in mastering spatial language due to the Chinese language system. For example, researchers found that Chinese children acquired spatial location words following the order of “inside, up, down, outside, back, front, middle, side, left, right” (Zhang, 1986; Kong and Wang, 2002). The use of spatial reference systems varies across different cultures and might stem from different spatial awareness. Some languages tend to involve self-centered (e.g., left and right) encoding positions, while other languages tend to involve concentric encoding positions (e.g., north and south) (Levinson, 2003). The concept of spatial orientation among the Han nationality in China is mainly based on the reference structure of “all things are one, and man and nature are one” (Zhu, 2017). Language and culture have crucial influences on the development of children’s spatial concepts and spatial language in different societies. Currently, the relevant research is mainly focused on research on a particular type of spatial language (e.g., spatial locations and dimensions). It is necessary to analyze young children’s use of different types of spatial language in the kindergarten context.

Recently, there has been increasing evidence that spatial language contributes to the development of spatial skills (Miller et al., 2016). Several studies have shown that the development of children’s spatial skills is directly affected by the spatial language environment created by adults for children, such as adults’ spatial words in free-play environments (Pruden et al., 2011), parent-child relationships (Levine et al., 2012) and family social and economic levels (Verdine et al., 2014). In addition to family environmental factors, the development of children is different depending on age and gender. The level of development of young children’s ability to understand spatial representation language at the age of 3–5 is significantly higher than their ability to use spatial representation language (Pang et al., 2008). Otherwise, there were no sex differences in children’s performance in the WPPSI-III Block Design subtest or the Spatial Analogies task. However, the cumulative spatial tokens of children showed a marginally significant difference in the amount of spatial language used by boys and girls (Pruden et al., 2011). The use of spatial language by children of different ages and genders in the kindergarten classroom environment needs to be studied further.

### Relationship Between Block Play and Spatial Language

In recent years, there have been many studies on spatial language. Some studies have investigated children’s representational ability to understand spatial language in the form of researchers’ commanding children to put objects in certain places, asking them to also find and describe places. Loewenstein and Gentner (2005) provided clues about spatial language abilities in 4-year-old children. They found that spatial language clues could help them complete tasks more effectively. Children are better at producing spatial language (e.g., left/right, pass/side, or middle) related to tasks (Hermer-Vazquez et al., 2001; Ankowski et al., 2012; Miller et al., 2016). The current research mainly explores the relationship between providing spatial clues for children and their spatial language development in the task. However, in free play, other situations might also provide effective spatial clues for children, and the relationship between the situation and spatial language requires further study.

Blocks are basic materials used by children to construct and represent the world around them during play (Pan et al., 2016). Children need to think about the choice of the shape and size of blocks, the adjacent relationship of orientation, the stability of building works, all of these require children to have an ability to mobilize space comprehensively (Wu et al., 2019). During block building, children perceive and learn about the intrinsic features of objects, such as how objects vary with dimensions of size, pattern, symmetry, and shape (Casey and Bobb, 2003; Verdine et al., 2014; Suh et al., 2019). They can perceive space, geometry, and correctly grasp the concept of space (e.g., “Where am I?” “How far am I from it?” “Where is it?”) (Clements et al., 1997; Hu, 2018). Zhang (2013) and Kang et al. (2020) measured the spatial skills and building ability of children who received pretest and posttest in building training, the same conclusion was that block play helped to improve children’s spatial skills. Several studies have provided suggestive evidence that early block building can promote the development of children’s spatial thinking (Verdine et al., 2014; Simoncini et al., 2020).

Blocks are also the media for children’s original ideas and life experience, with an open versatility that means they can be and re-created. They provide children with a representation transformation mechanism to help them better explore the world (Hu, 2018). Block play provides opportunities for children’s language learning and communication. Young children effectively use oral language and communicate with their peers (Cheng, 2017), express their construction goals and ideas, and naturally generate relevant spatial language. Ferrara et al. (2011) found that the frequency of children’s spatial language in a common interactive group is lower than that in a block play group, which indicated that block play could stimulate children’s conversation about spatial concepts, such as spatial orientation and matching the shape of blocks.

### The Relationship Between Building Blocks, Language and Spatial Representation

Spatial representation describes the form of an object’s position and spatial relation in individual psychology, and the internal process of individual thinking, reasoning, and the operation of spatial information (Zhao, 2006). The solution to spatial problems can be inextricably linked to the participation of spatial representation. As one of the crucial aspects of spatial cognition, an ability to understand and use spatial representation plays an important role in the process of exchanging and manipulating spatial information (Pang et al., 2008). Studies have shown that exposure to spatial language and that when diverse contexts promote children’s spatial thinking. With stronger spatial representations, children may be able to dedicate more cognitive resources to spatial processing (Casasola et al., 2020). However, in the domain of spatial development, similar interactions among cognitive processes could underlie the spatial relations (Miller and Simmering, 2018). By analyzing the use of spatial language in building blocks, this study further develops understanding of children’s spatial representation, exploring the links between the representation of building blocks and linguistic representation of verbal communication in children’s cognitive spatial relations.

Building blocks are a representation of space. According to the study of Liu (2015, p. 568), when young children put blocks together, they can experience “proximity.” Sequentially, arranging the blocks produces the “sequence.” A certain space is composed of blocks to make the difference between “inside” and “outside.” Blocks are inverted, converted, and built to form a certain model and generate various spatial structures. The formation of spatial concepts essentially lies in “being experienced” rather than “being informed.” A building block is a highly practical spatial operational activity, providing rich opportunities for children to explore space, enabling them to directly and concretely perceive and experience abstract spatial relations. Moreover, building blocks provide children with a diversity and amount of spatial labels that may promote the representation of children’s spatial information on a broader level than simply supporting labels for spatial information (Casasola et al., 2020). Further research has suggested that experience of spatial activities in block building may improve selective attention in children (Miller and Simmering, 2018). Specifically, children who play more spatial games tend to perform better in spatial performance, which indicates that they may learn how to focus on relevant information through spatial play (Jirout and Newcombe, 2015; Miller and Simmering, 2018).

Verbal communication during the process of building blocks facilitates the linguistic representation of space. Plumert and Hawkins (2001) have suggested that children aged 4–5 years old are able to understand the representational relationship between spatial language and spatial relationships in reality. For children, space is an abstract and difficult concept, while language is an effective tool to help children understand the concept of space. Multiple studies have proved that language plays a key role in spatial development through creating spatial labeling, changing spatial representations, and directing attention/encoding (Gentner, 2003, 2016; Dessalegn and Landau, 2008; Miller et al., 2016; Miller and Simmering, 2018). However, not all languages can promote the development of children’s spatial skills. Children may not spontaneously recognize and produce spatial information about location before being prompted, and knowledge of language alone is insufficient to explain children’s spatial performance (Farran and O’ Leary, 2016; Miller and Simmering, 2018). At this time, verbal communication with peers can be employed as an external linguistic representation to prompt, express, transmit, and memorize spatial information and participate in the encoding and processing of children’s spatial relations.

One possibility is that verbal communication can attract children’s attention to relevant spatial information, improve children’s selective attention, and stimulate children to produce language related to location information. As studies have suggested, “when children are provided with language cues by an adult, the language can direct their attention to improve their spatial performance (Miller and Simmering, 2018).” Similarly, children often produce location terms when prompted by peers in the contexts of block play. Verbal communication with peers makes it possible for children to focus attention on the labeled spatial information, improve the understanding of how children use particular spatial words differently based on the context and enhance their ability to use task-relevant adaptive language. Another possible explanation is that hearing and expressing the relational language in verbal communication promotes the development of children’s representational structure, thus promoting children’s spatial thinking process. As for the effects of acquiring and using spatial language within a language community, Loewenstein and Gentner (2005) suggested that “once relational terms have been acquired, hearing relational language might facilitate encoding relations in ways consistent with the semantics of the terms.” Thus, “hearing the spatial language induces a conceptual representation of spatial relations.” They also observe that, “relational labels invite the child to notice, represent, and retain structural patterns of elements (Gentner and Loewenstein, 2002, p. 103).” Relational language provides representational tools with which speakers can create construals that facilitate reasoning (Gentner and Loewenstein, 2002; Loewenstein and Gentner, 2005).

The gestures of parents during spatial conversations could predict children’s spatial language, which may also be involved in children’s future spatial cognition (Pruden et al., 2011). In the process of peer communication, children tend to use active representation to assist the expression of spatial information and spatial relations, and the overlapping of language representation and active representation occurs in the process of spatial representation.

Overall, building blocks and verbal communication are imperative forms for children to understand and use spatial representation. Children can determine the location of target objects according to the linguistic representation requirements of others and the need for building models, so as to understand the spatial relationship. Children use language, model operation, active, and other representational forms to convey spatial information to others. They extract and organize representational symbols to communicate and spread spatial information through verbal and action communication in peer interaction. Casasola et al. (2020) proved that providing spatial language as children manipulate blocks makes it possible for children to align their actions and attention to the labeled spatial information. The co-occurrence between building blocks and verbal communication may have created a synergy that is pitched to bolster the effect of spatial labels on children’s spatial thinking. Therefore, exploring verbal communication with peers whilst using building blocks could help us to further understand the synergy between different forms of spatial representation and explore the relationship between language and spatial cognition.

### The Present Study

Since block play embodies and promotes children’s spatial skills and spatial language, it provides a context to study the development of children’s spatial skills and spatial language. We examined the use of spatial language during block play in 228 children from the younger, middle, and older age groups, to examine the features and related factors of young children’s spatial language. The questions we examined are described below.

First, what types of spatial language do young children use during block play? Previous empirical evidence shows that spatial skills are positively correlated with block building skill (Zhang, 2013; Kang et al., 2020). The spatial skills and spatial language of children might be inspired by peer interaction (Cheng, 2017). Therefore, the content and frequency of young children’s spatial language use might vary in different contexts of block play. In contexts with more complex construction structures and more peer interactions, children might more frequently use spatial language in complex forms and contents.

Second, how does the use of spatial language during block play vary with age and gender? Previous studies have shown that children who were 3–5 years old could comprehend spatial language better than they could use it (Pang et al., 2008). There are also some differences in the spatial language used by children of different genders (Chan, 2007). Children of different ages and genders use different types of spatial language during block play.

## Materials and Methods

### Participants

Considering the influence of daily experiences in play, four kindergartens were selected to provide medium-sized wooden blocks in the classroom and conduct free play every day. The four kindergartens had the same (Ji 级) and category (Lei 类), and these kindergartens were often called R1C1 kindergartens (this meant that kindergartens of the top rank and category were regarded as the best) (Pan et al., 2010). In the classroom, young children were randomly selected (*n* = 228, 114 boys and 114 girls) in a total of 57 groups: 19 groups in younger class (*n* = 76, mean age = 50.99 months, range: 41–59 months, SD = 4.17), 20 groups in the middle age class (*n* = 80, mean age = 60.98 months, range: 46–71 months, SD = 5.68), and 18 groups in older class (*n* = 72, mean age = 69.19 months, range: 62–76 months, SD = 3.80).

The children in the study came from the same racial backgrounds, and they could communicate well with their peers and express their ideas using Mandarin. All the kindergarten classrooms were based on developmentally appropriate early childhood practices (Casey et al., 2008), with a variety of activity centers in the rooms (including a block area), and choice time for the children to play in these areas (e.g., constructive play, role play, and exhibition play). The researchers made sure that there were a sufficient number of blocks of different sizes and shapes provided in each of the classrooms.

### Material

#### Material, Size, Shape, and Quantity of Blocks

Medium-sized wooden blocks were chosen. The size of the unit blocks was 3.5 cm × 7 cm × 14 cm, including 18 types of shapes (e.g., cuboid, cylinder, slope, triangle, and Y-shape) formed based on the size of the unit block. As the number of blocks was reduced, it had a significant impact on the level of children’s construction (Yang et al., 2020). Under the condition that the number of pairs of blocks (such as isosceles right triangle blocks or slope blocks) was even, according to the number and use of different shapes of blocks by the children, we ensured that the number of blocks the children has access to was greater than 200.

### Design

#### Play Partners and Zone Area

In the classroom, a large meeting room, or a music classroom, we created the building block play area. The number of young children entering the block area, as specified by most classrooms in kindergarten practice, did not exceed six. In most cases there were four children (Pan et al., 2016), and a space density of 1.47 square meters was an ideal activity site (Zhang and Fang, 2018). As mentioned, the number of young children in the same play group was limited to four, and the per capita activity area was 1.5 square meters.

#### Play Duration

Young children were allowed to enter the block area for free play. The duration was from the time when the children began constructing to the time when they stopped constructing, proceeded to other types of activities for a long time, and did not return to playing blocks. The average time for block play in this experimental study was 25 min.

### Procedure

Each play consisted of two boys and two girls randomly selected by kindergarten teachers from the same classroom. With no other children on-site, the young children entered the block area for free play. Before the children entered the area, the researchers informed them of the basic rules of behavior, such as not throwing blocks and not constructing directly beside the block cabinet. The researchers did not intervene unless the children’s behavior may have threatened their physical safety. Children were allowed to introduce their building work when the play was over. The researchers videotaped the entire process and took pictures of the young children’s construction structure during the building process. In this study, 57 videos and several pictures of children’s block play were collected.

### Coding

#### Coding Spatial Language of Young Children

Based on studies by Ferrara et al. (2011) and Levine et al. (2012), the present study divided young children’s spatial language into (1) spatial locations (up and down), (2) deictic terms (here and there), (3) dimensions (long and tall), (4) spatial features or properties (curvy and straight), (5) shapes (rectangle and square), and (6) spatial orientations or transformations (“turn it around,” “the man is facing the block”). We transcribed all language during the free block play, coded the spatial locations, deictic terms, dimensions, shapes, spatial features or properties, spatial orientations and transformations of each child during play, and counted their frequency. Words with metaphorical meaning (e.g., “he sits on the ground,” “block this up”) were temporarily not considered. In the same sentence, spatial language expressed with the same meaning was counted once. Considering the differences between the English and Chinese languages, we listed English-speaking and Chinese-speaking coding tables, as shown in Table 1.

**TABLE 1:** Categories of spatial language in English-speaking and Chinese-speaking.

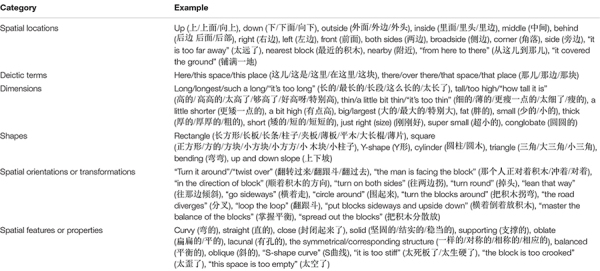

#### Coding Construction Structure of Young Children

Researchers evaluated children’s building skills when constructing a structure and the spatial structure of blocks (Hanline et al., 2001; Casey et al., 2008; Ramani et al., 2014). As Borriello and Liben (2017) said, “complexity was judged by the number of blocks, the number of horizontal levels and vertical planes, and the extent to which all blocks were visible.” We assessed the construction structure completed by each child. Based on the complexity of the works (Casey et al., 2008; Pan et al., 2016), children’s construction structures were divided into seven levels: (1) random block placement, (2) tile/pile structure, e.g., one-dimensional structure (row of single blocks, or stack of single blocks), or two-dimensional structure (no internal space), structure with no width (a wall), no height (a floor), or no length (a two block-wide tower), (3) simple overhead structure, e.g., two-dimensional structure vertical internal space (arches), (4) crowd around structure, e.g., two-dimensional with horizontal internal space (enclosure), (5) complex overhead structure, e.g., three-dimensional structure vertical internal space (house), three blocks high and above, the structure of each layer are different, (6) simple combination structure, e.g., two-dimensional vertical or horizontal internal space plus depth to make a three-dimensional structure (arch + 1 or more blocks placed in front or behind, or two walls), (7) complex combination structure, complex overhead structure + horizontal internal space plus depth (or crowd around structure etc.) to make a three-dimensional structure. Each construction structure completed by the children during block play was coded and scored (0–6 points in sequence).

#### Coding Building Form of Young Children

According to the level of children’s social interaction behavior (Ma et al., 2013; Hu, 2018), the children were free to choose whether to cooperate with their peers during play. Block building forms were divided into independent construction and cooperative construction.

## Results

### Descriptive Statistics

Generally, young children used spatial locations, deictic terms, and dimensions more frequently in block play, accounting for 76.38% of usage. Young children used spatial locations most frequently (more than 30%). Next, the proportion of spatial deictic terms (22.56%) and dimensions (22.19%) was quite similar. Then, shapes account for 11.62%, while spatial orientations or transformations (6.81%) and spatial features or properties (5.20%) occur relatively less frequently, with a total of only 12.01% (see Table 2).

**TABLE 2:** Descriptive statistics of spatial language.

	MAX	*M*	SD	Proportion (%)	Total
Spatial locations	47	4.91	6.16	31.63	1119
Deictic terms	21	3.50	4.15	22.56	798
Dimensions	25	3.44	3.80	22.19	785
Shapes	13	1.80	2.38	11.62	411
Spatial orientations or transformations	7	1.06	1.63	6.81	241
Spatial features or properties	8	0.81	1.29	5.20	184
Spatial language	94	15.52	15.60	100.01	3538

*For the rounding-off method, the sum is 100.01%. For the minimum value is “0,” it does not show up in the table.*

The first result of the present study relates to the spatial position words used by children, which were more diversified. According to different directions and areas, spatial locations were divided into vertical direction (e.g., up and down), horizontal direction (e.g., left, right, nearby, side, front, and behind), specific region (e.g., corner, edge, and spatial common sense), relative distance (e.g., side), and dynamic position (e.g., cross, leave, around, and enter), and then classified statistics were conducted (Bracken and Crawford, 2010; Zhu, 2017). Young children were more inclined to use spatial language in the vertical direction (34.50%) and dynamic position (31.99%), while the horizontal direction (16.00%), specific region (11.35%), and relative distance (6.17%) were used less frequently (see Table 3).

**TABLE 3:** Descriptive statistics of spatial direction locations.

	MAX	*M*	SD	Proportion (%)	Total
Vertical direction	31	1.69	3.16	34.50	386
Dynamic position	17	1.57	2.26	31.99	358
Horizontal direction	6	0.79	1.28	16.00	179
Specific region	10	0.56	1.32	11.35	127
Relative distance	4	0.30	0.71	6.17	69
Spatial locations	47	4.91	6.16	100.01	1119

*For the rounding-off method, the sum is 100.01%. For the minimum value is “0,” it does not show up in the table.*

The second finding was that young children tended to use deictic terms with strong functionality and directionality. Usually, words such as “here” (这儿/这是/这里/在这里) and “there” (那儿/那边/那里) were used to represent the space area where the object was located, words such as “where” (哪里) were used to ask for the spatial location of the object, and words such as “this space/this place” (这块/这片), “that space/that place” (那块/那片) were used to delimit the spatial scope. Moreover, young children often used spatial locations along with gesture language. They tended to use gestures to divide the space and point to the region represented.

The third finding was that, among the shapes, tetragon words accounted for the highest proportion (34.30%). Specifically, young children could use relatively standard shape words, including “triangle” (三角形), “ellipse” (圆形), “semicircle” (半圆形), “rectangle” (长方形), and “square” (正方形), which to represent the shape of objects (accounting for 58.87%). Among them, the frequencies of “large and small triangle” words (29.68%) were the highest, “ellipse and semicircle” words (19.22%) were the second most frequent, and “rectangle” words (5.35%) and “square” words (4.62%) were the lowest. However, when young children used shape words, they often replaced shape words with object’s names (accounting for 41.12%). Furthermore, the children used similar things they experienced in daily life to represent all kinds of blocks with different shapes. Most of them used “column” (圆柱) (13.38%) to represent cylinder blocks, “long strip (长条), long block (长木), long board (长板), flat plate (平板), thin sheet (薄片)” (13.38%) to represent cuboid blocks, “boxes, small boxes” (方块/木块/小木块/小方方/小方块) (10.95%) to represent square blocks, “trapezoid, up and down slope” (梯形/上下坡) to represent oblique triangle blocks, and “Y-shaped, curved” (Y 形/拐弯) to represent irregular-type blocks (3.41%) (see Table 4).

**TABLE 4:** Descriptive statistics of shapes.

Shapes	Representation words	Count	Proportion (%)
Triangle	Big triangle, small triangle	122	29.68
Cylinder	Ellipse, semicircle	79	19.22
	Column	55	13.38
Tetragon	Long strip, long block, long board, flat plate, thin sheet, etc.	55	13.38
	Boxes, small boxes, etc.	45	10.95
	Rectangle	22	5.35
	Square	19	4.62
Others	Trapezoid, up and down slope, Y-shaped, curve, etc.	14	3.41

*For the rounding-off method, the sum is 99.99%.*

The fourth result was that spatial orientations or transformations (6.81%) and spatial features or properties (5.20%) were used less frequently. In the process of building, young children mainly used spatial language such as “turn” (转过来), “go straight”(直走), “on end”(竖起来), “turn around”(翻转/掉头) “circle around”(围起来), and “turn the blocks around” (拐弯) to represent the change of the blocks and the movement direction of the building. They attempted to use spatial language such as “facing” (正对/面向) and “lean that way” (往那边倾斜) to describe the spatial position relationship and represent spatial positioning information.

Finally, we also found that the children mainly used spatial language (e.g., big, small, long, and high) to perceive the spatial dimension, and used the words “curvy” (弯曲的/弯的), “straight” (直的), “empty” (空的), “stable” (稳固的), “oblique” (斜的) to describe the spatial features or properties of the building. Similarly, young children had an emotional tendency in using words for the dimensions and spatial features or properties, showing their tone of praise, wonder or complaint. For instance, the words such as “it is too high” (太高了/够高了/特别高), “it is too stiff” (太死板了). In addition, young children used comparative and superlative words such as “biggest” (最大的), “a little shorter” (更短的). Interestingly, the use of dimension words was also characterized by personification, and children would use words describing people (thin, fat, short, etc.) to represent the size of objects. Furthermore, young children were able to use more complex characterizations of spatial features or properties, such as “symmetrical/corresponding” (一样的/匀称的/对称的), “balanced” (平衡的), and lacunal (有孔的).

### Block Building Context

The block building context mainly included construction structures and forms made by the children. Firstly, to analyze the frequency of children’s spatial language for different construction structures and based on the spatial dimensionality and hierarchical integration of the children’s construction structure, we split them into three levels. The lower construction structure included random block placement and tile/pile structure. The middle construction structure included a simple overhead structure and crowd around the structure. The higher construction structure included a complex overhead structure, simple combination structure, and complex combination structure. Next, according to the children’s choice as to whether they would cooperate with peers during building block play, the block building form was divided into independent construction and cooperative construction. Independent construction included the spatial language generated by young children’s self-talk.

The descriptive statistics in Table 5 show that the more complex the construction structure, the more spatial language children would use. The frequency of young children’s spatial language in cooperative construction was higher than during independent construction. Subsequently, we conducted a series of 3 (construction structure: lower, middle, and higher) × 2 (construction form: independent vs. cooperative) analysis of variance (ANOVA) tests to examine the differences between structure and form in spatial language. In these ANOVAs, construction structure and form were the between-subject variables, the frequency of spatial language and different types (dimensions, shapes, spatial features or properties, deictic terms, spatial locations, spatial orientations or transformations) were dependent variables. The results of the 3 × 2 ANOVAs indicated that the main effect of construction structure in spatial language was significant, *F*(2,225) = 7.65, *p* < 0.05, η^2^ = 0.064. The *post hoc* test proved that children who built higher construction structures used significantly more spatial language than those who built middle and lower construction structures (*p* < 0.05). The main effect of construction form in spatial language was also significant, *F*(2,225) = 18.88, *p* < 0.001, η^2^ = 0.078, with more spatial language in cooperative construction than independent construction.

**TABLE 5:** Descriptive statistics of construction structure and form.

	Construction structure						Construction form			
		
	Lower (A) (*N* = 54)		Middle (B) (*N* = 98)		Higher (C) (*N* = 76)		Independent (*N* = 107)		Cooperative (*N* = 121)	
					
	*M*	SD	*M*	SD	*M*	SD	*M*	SD	*M*	SD
Dimensions	2.93	2.92	2.97	3.82	4.42	4.14	2.23	2.74	4.51	4.26
Shapes	0.80	1.17	2.07	2.92	2.17	2.04	0.93	1.68	2.58	2.63
Spatial features or properties	0.61	1.12	0.47	0.92	1.38	1.59	0.48	0.92	1.10	1.49
Deictic terms	2.50	3.03	2.60	3.53	5.37	4.92	2.30	3.01	4.56	4.70
Spatial locations	2.98	3.07	3.76	4.72	7.76	8.16	3.31	3.94	6.32	7.33
Spatial orientations or transformations	0.85	1.64	0.83	1.45	4.42	4.14	0.64	1.12	1.43	1.90
Spatial language	10.67	9.87	12.69	13.97	2.17	2.04	9.88	10.05	20.50	17.82

*“A” stands for lower construction structure, “B” for middle construction structure, and “C” for higher construction structure.*

In practical terms, the main effect of construction structure in shapes was significant, *F*(2,225) = 5.51, *p* < 0.01, η^2^ = 0.047. The main effect of construction structure in spatial features or properties was significant, *F*(2,225) = 7.81, *p* < 0.01, η^2^ = 0.066, The main effect of construction structure in deictic terms was significant, *F*(2,225) = 7.42, *p* < 0.01, η^2^ = 0.063. The main effect of construction structure in spatial locations was significant, *F*(2,225) = 8.28, *p* < 0.001, η^2^ = 0.069. The *post hoc* test proved that the children who built higher construction structure used spatial features or properties, deictic terms and spatial locations were significantly more than those built middle and lower construction structure (*p* < 0.05). Shapes occurred significantly more often among children who built higher and middle construction structure than those built lower construction structure (*p* < 0.05). Moreover, the main effect of construction form in dimensions was significant, *F*(2,225) = 16.09, *p* < 0.001, η^2^ = 0.068. The main effect of construction form in shapes was significant, *F*(2,225) = 22.66, *p* < 0.001, η^2^ = 0.093. The main effect of construction form in spatial features or properties was significant, *F*(2,225) = 7.34, *p* < 0.01, η^2^ = 0.032. The main effect of construction form in deictic terms was significant, *F*(2,225) = 10.86, *p* < 0.01, η^2^ = 0.047. The main effect of construction form in spatial locations was significant, *F*(2,225) = 7.70, *p* < 0.01, η^2^ = 0.035. The main effect of construction form in spatial orientations or transformations was significant, *F*(2,225) = 9.51, *p* < 0.01, η^2^ = 0.041. Children who adopted cooperative construction had a higher frequency of each type of spatial language than those with independent construction. No significant interaction effect was observed between construction structure and form (*p* > 0.05) (see Table 6).

**TABLE 6:** Comparison of differences among children of different construction structure and form (*N* = 228).

		df	MS	*F*	*p*	η^2^	*Post hoc*
Dimensions	Structure	2	29.35	2.25	0.108	0.020	n.s.
	Form	1	209.74	16.09***	0.000	0.068	
	Structure × form	2	14.05	1.08	0.342	0.010	
Shapes	Structure	2	26.45	5.51**	0.005	0.047	B > A, C > A
	Form	1	108.84	22.66***	0.000	0.093	
	Structure × form	2	6.57	1.37	0.257	0.012	
Spatial features or properties	Structure	2	11.46	7.81**	0.001	0.066	C > A, C > B
	Form	1	10.77	7.34**	0.007	0.032	
	Structure × form	2	0.90	0.61	0.543	0.005	
Deictic terms	Structure	2	111.32	7.42**	0.001	0.063	C > A, C > B
	Form	1	162.99	10.86**	0.001	0.047	
	Structure × form	2	7.58	0.51	0.604	0.005	
Spatial locations	Structure	2	273.81	8.28***	0.000	0.069	C > A, C > B
	Form	1	263.50	7.70**	0.005	0.035	
	Structure × form	2	31.79	0.51	0.604	0.005	
Spatial orientations or transformations	Structure	2	4.52	1.82	0.164	0.016	n.s.
	Form	1	23.58	9.51**	0.002	0.041	
	Structure × form	2	0.97	0.39	0.677	0.004	
Spatial language	Structure	2	1559.63	7.65**	0.001	0.064	C > A, C > B
	Form	1	3850.45	18.88***	0.000	0.078	
	Structure × form	2	17.76	0.09	0.917	0.001	

n.s. *p* > 0.05, *p < 0.05, **p < 0.01, ***p < 0.001.

*“A” stands for lower construction structure, “B” for middle construction structure, and “C” for higher construction structure.*

### Age and Gender Difference

The descriptive statistics in Table 7 showed that the frequency of children’s spatial language increases with the growth of age. In the study, we conducted a series of 3 (age class: younger class, middle class, and older class) × 2 (gender: boy vs. girl) ANOVA tests to examine age class and sex differences in spatial language. In these ANOVAs, age class and gender were the between-subjects variables, the frequency of spatial language and different types (dimensions, shapes, spatial features or properties, deictic terms, spatial locations, spatial orientations or transformations) were the dependent variables. Results of the 3 × 2 ANOVAs showed that the main effect of age class in spatial language was significant, *F*(2,225) = 6.84^∗∗^, *p* < 0.01, η^2^ = 0.058. The *post hoc* test proved the spatial language of children in the older class was significantly higher than that in the younger class (*p* < 0.05). Concretely, the age class main effect of in spatial features or properties was significant, *F*(2,225) = 5.51^∗∗^, *p* < 0.01, η^2^ = 0.047. The age class main effect of in deictic terms was significant, *F*(2,225) = 13.37, *p* < 0.001, η^2^ = 0.107. The age class main effect of in spatial locations was significant, *F*(2,225) = 6.00, *p* < 0.01, η^2^ = 0.051. The age class main effect of in spatial orientations or transformations was significant, *F*(2,225) = 3.78, *p* < 0.05, η^2^ = 0.033). The *post hoc* test proved the spatial features or properties, spatial locations and spatial orientations or transformations of children in the older class was significantly higher than that in the younger class (*p* < 0.05), deictic terms of children in the older and middle class was significantly higher than that in the younger class (*p* < 0.05). Results showed no gender differences in the spatial language of children, but there was a marginally significant difference in the number of shapes used by boys and girls (*p* = 0.067). No significant interaction effect was observed between age class and gender (p > 0.05) (see Table 8).

**TABLE 7:** Descriptive statistics of age class and gender.

	Age class						Gender			
		
	Younger (Y) (*N* = 76)		Middle (M) (*N* = 80)		Older (O) (*N* = 72)		Boy (*N* = 114)		Girl (*N* = 114)	
					
	*M*	SD	*M*	SD	*M*	SD	*M*	SD	*M*	SD
Dimensions	3.32	3.68	3.41	4.28	3.61	3.35	3.23	3.77	3.66	3.81
Shapes	1.33	2.31	1.95	2.64	2.14	2.08	1.52	2.06	2.09	2.64
Spatial features or properties	0.55	1.04	0.69	1.12	1.21	1.59	0.89	1.44	0.72	1.13
Deictic terms	1.84	2.70	3.55	3.90	5.19	4.96	3.41	4.16	3.59	4.16
Spatial locations	3.30	4.77	4.79	5.58	6.74	7.50	4.76	6.36	5.05	5.97
Spatial orientations or transformations	0.66	1.09	1.16	1.86	1.36	1.76	1.01	1.66	1.11	1.60
Spatial language	11.00	12.86	15.55	15.19	20.25	17.37	14.82	15.84	16.21	15.39

*“Y” stands for younger class, “M” for middle class, and “O” for older class.*

**TABLE 8:** Comparison of differences among children of different age class and gender (*N* = 228).

		df	MS	*F*	*p*	η^2^	*Post hoc*
Dimensions	Age class	2	1.67	0.12	0.892	0.001	n.s.
	Gender	1	10.67	0.73	0.392	0.003	
	Age class × gender	2	10.06	0.69	0.502	0.006	
Shapes	Age class	2	13.47	2.45	0.088	0.022	n.s.
	Gender	1	18.64	3.39	0.067	0.015	
	Age class × gender	2	9.76	1.78	0.172	0.016	
Spatial features or properties	Age class	2	8.83	5.51**	0.005	0.047	O > Y
	Gender	1	1.71	1.07	0.303	0.005	
	Age class × gender	2	1.20	0.75	0.475	0.007	
Deictic terms	Age class	2	207.91	13.37***	0.000	0.107	M > Y, O > Y
	Gender	1	2.32	0.15	0.7	0.001	
	Age class × gender	2	18.19	1.17	0.312	0.01	
Spatial locations	Age class	2	218.83	6.00**	0.003	0.051	O > Y
	Gender	1	4.95	0.14	0.713	0.001	
	Age class × gender	2	33.68	0.92	0.399	0.008	
Spatial orientations or transformations	Age class	2	9.83	3.78*	0.024	0.033	O > Y
	Gender	1	0.60	0.23	0.632	0.001	
	Age class × gender	2	1.24	0.48	0.622	0.004	
Spatial language	Age class	2	1581.82	6.84**	0.001	0.058	O > Y
	Gender	1	116.62	0.50	0.478	0.002	
	Age class × gender	2	308.48	1.33	0.265	0.012	

n.s. *p* > 0.05, *p < 0.05, **p < 0.01, ***p < 0.001.

*“Y” stands for younger class, “M” for middle class, and “O” for older class.*

### Correlations

We performed two-tailed Pearson and Spearman correlation of variables to determine the relationship among variables. As shown in Table 9, young children who built construction structures were significantly related to spatial language (*r* = 0.321, *p* < 0.01). Building a complex structure mobilized young children to use more spatial language. Specifically, there were significant positive correlations among the frequency of dimensions, shapes, spatial features or properties, deictic terms, spatial locations, spatial orientations or transformations, and construction structures built by young children (*r* = 0.171, *p* < 0.01; *r* = 0.292, *p* < 0.01; *r* = 0.302, *p* < 0.01; *r* = 0.286, *p* < 0.01; *r* = 0.288, *p* < 0.01; *r* = 0.239, *p* < 0.01). Next, there was a significant positive correlation between children’s choice of the building form in block play and their spatial language (*r* = 0.341, *p* < 0.01). The young children who adopted cooperative construction had significantly higher spatial language in shapes (*r* = 0.348, *p* < 0.01), dimensions (*r* = 0.301, *p* < 0.01), spatial positions (*r* = 0.245, *p* < 0.01), deictic terms (*r* = 0.273, *p* < 0.01), spatial orientations or transformations (*r* = 0.244, *p* < 0.01), spatial features or properties (*r* = 0.241, *p* < 0.01) than those who adopted independent construction. Therefore, young children who adopt the cooperative building form used more spatial language. Otherwise, there were a significant positive relation between young children’s age and spatial language (*r* = 0.289, *p* < 0.01). There was a significant positive correlation among the frequency of deictic terms, spatial locations, shapes, spatial features or properties, spatial orientations or transformations, and the age class of young children (*r* = 0.349, *p* < 0.01; *r* = 0.275, *p* < 0.01; *r* = 0.247, *p* < 0.01; *r* = 0.224, *p* < 0.01; *r* = 0.169, *p* < 0.05).

**TABLE 9:** Correlations among the variables (*N* = 228).

	1	2	3	4	5	6	7	8	9	10	11
1. Dimensions	–										
2. Shapes	0.615**	–									
3. Spatial features or properties	0.526**	0.345**	–								
4. Deictic terms	0.527**	0.391**	0.493**	–							
5. Spatial locations	0.588**	0.576**	0.602**	0.655**	–						
6. Spatial orientations or transformations	0.474**	0.314**	0.516**	0.526**	0.548**	–					
7. Spatial language	0.802**	0.695**	0.686**	0.808**	0.907**	0.666**	–				
8. Construction structure	0.171**	0.292**	0.302**	0.286**	0.288**	0.239**	0.321**	–			
9. Building form	0.301**	0.348**	0.241**	0.273**	0.245**	0.244**	0.341**	0.132*	–		
10. Age class	0.066	0.247**	0.224**	0.349**	0.275**	0.169*	0.289**	0.447**	0.209**	–	
11. Gender	0.057	0.120	–0.068	0.021	0.024	0.030	0.045	–0.105	0.132*	0.000	–

Gender, age class, and building form are the dummy variable: girl = 1, boy = 0; younger class = 1, middle class = 2, older class = 3; cooperative form = 1, independent form = 0. *p < 0.05, **p < 0.01.

## Discussion and Conclusion

The purpose of this study was to explore the frequency, type, and level of spatial language in the context of block play and the differences that vary by age and gender in young Chinese children. Overall, spatial locations, deictic terms, dimensions, and shapes were used more frequently by young children, and spatial features or properties and spatial orientations or transformations were used less frequently. Specifically, the following conclusions were drawn: (a) spatial locations were used most frequently, and young children tended to use vertical locations to represent the corresponding location; (b) most young children used gesture in conjunction with spatial deictic terms; (c) tetragon words were more frequently used in the shape words, and the representation of shapes showed alternatives, collective tendencies, and gender differences; (d) the frequency of spatial language in children was related to their construction structure and form; and (e) the age class of young children was also associated with the frequency of spatial language.

One important finding from the present research was that the most frequent use of spatial language during young Chinese children’s block play involved spatial locations, which accounted for nearly a third of spatial language. These results agree with prior findings that English-speaking children acquired many spatial relational terms in preschool years, and they use the most spatial position words in free block play (Ferrara et al., 2011). Three-year-old children have shown high levels of comprehension for these basic spatial terms such as “on, in, and under and top, middle, and bottom” (Meints et al., 2002; Loewenstein and Gentner, 2005). Moreover, young Chinese children’s spatial locations appeared in the order of vertical direction, dynamic position, horizontal direction, a specific region, and relative position from high to low. They preferred to use spatial language in the vertical direction (e.g., “up,” “down”). The general order of spatial locations was the same as previous results. Preschool is a period when young children most rapidly master spatial locations. From the age of three years old, Chinese-speaking children identify spatial orientation according to the development order of “up/down-front/back-left/right” (Huang, 2007, p. 209–211). One possible reason involved the spatial properties of blocks. Blocks occupied a certain space in both vertical and horizontal directions. The size of the space occupied by a block in the vertical or horizontal direction depends on the way it is placed. Blocks could make a building higher when they are stacked together, head-to-tail connection could make the building longer, and continuous tiling could make the area occupied by objects continue to expand (Liu, 2015, p. 565–571). Young children use blocks to construct all kinds of buildings to represent the world. Through the analysis of young children’s construction structure in free block play, we found that the themes of structures were mainly houses, bridges, and roads (Yang et al., 2020). Most structures adopted a vertical construction to form a simple combination, complex overhead, and complex combination structure. Therefore, the frequency of use of vertical direction words was higher. In addition, young Chinese children used a variety of dynamic location words to represent changes in spatial positions. Words such as “let us go through here” (从这儿钻过去), “enter into” (进入), and “get out” (离开) reflected the interaction between them and the spatial structure of buildings, words such as “step over” (踩过去/跨过去), “walk around” (绕过去), and “pass through” (穿过) indicated the way they used limited space, and words such as “move past” (从这里过去) and “put back” (回来) showed their perception of the spatial distance between themselves and blocks.

We also found that young Chinese children used spatial deictic terms with strong functionality and directionality, aiming to express precise spatial locations through language. The use of deictic terms indicated that young children could understand the building space occupied by blocks, structure, and spatial relationships. Spatial deictic terms were often used to represent the spatial location of blocks and different spaces. They could be used to help young children better plan spatial scope, by developing consciousness of spatial matching, clarifying the space occupied by the building [such as using “this space” (这片/这块) or “that space” (那片/那块) to delimit the spatial area], and coordinate a continuation of the same space. Of course, the division of space also reflected children’s competition for limited space. Whether this involved negotiation or competition about space, it consistently reflected their spatial awareness in the process. Children had a certain understanding of the spatial structure, the spatial location of a structure, and the space occupied by humans. The use of spatial language showed the differentiation of “the relationship of object and I.” Young children began to distinguish and think about the spatial location of the “object” and “I,” which could also help children “decentrate” to some extent and promote their social development. However, when young children expressed spatial properties, the effect of their expression was not satisfactory due to their limited spatial vocabulary. Therefore, when young children used spatial deictic terms, they made full use of gesture language and other actions to assist in representing the space area and scope, pointing or delineating the space. The role of gesture language was emphasized in both Chinese and English children’s spatial language. Gesture language with spatial information could not only help children and their peers understand linguistic information and improve the quality of communication but also promote the encoding of spatial information (Alibali, 2005; Cartmill et al., 2010; Li and Kang, 2019). One study showed that the amount of young children’s spatial language was positively correlated with the number of adult’s gesture and spatial language, and gesture language was an important predictor of young children’s spatial language when controlling adults’ spatial language (Young et al., 2014). Li and Kang (2019) proposed that gesture language conveyed spatial concepts to young children in a vivid way, and spatial concepts would be understood, transmitted, and shared by peers. Therefore, young children could be encouraged to express spatial language in two ways: gesture language and oral language. When adults help young children input and output spatial language, they should try their best to use oral language and gesture language.

Our findings showed that the representation of tetragon words accounted for the highest proportion, among the shapes words. In the previous literature, young Chinese children showed a preference for tetragon blocks (Sun, 2015), they tended to use rectangular blocks to represent the main part of a building (Yang et al., 2020). Normally, young children used more tetragon words to represent the shapes of blocks. In the study, young children could distinguish squares, triangles, and other shapes and use standard shape words to represent the shape of blocks, which also conformed to previous studies indicating that young children older than 4 years old could completely recognize Euclidean figures (e.g., triangle, square, rectangle) (Zhao, 2007). Otherwise, young children used the names of similar objects and the use of objects to represent the shape of blocks. For example, the words “strip (长条), long board (长板)” and other similar objects were used to represent rectangular blocks, the word “column” (圆柱/圆木) was used to represent cylindrical blocks, irregular blocks used for turning were named “bending” (弯弯), and oblique triangular blocks were named according to the purpose of “up and down slope” (上下坡). Therefore, the representation of young children’s shapes showed an alternative. Based on their own life experience and building needs, young children creatively used shapes related to the theme and content of building blocks and used various symbols to represent the shapes of blocks, such as “small square” (小方块) and “slice” (薄片). These symbols could be spread among children in the same group, which promoted the transformation of the representation from “personal” to “collective” and ultimately reached a consensus (Liu, 2015, p. 567–581). Thus, the representation of young children’s shapes had the meaning of “communication,” showing the tendency toward collectivization within small groups.

We had two major findings relating to the block building context. The first is that young Chinese children who built higher construction structures used significantly more spatial language than those who built middle and lower construction structures. Many studies have proved that there was a positive correlation between children’s building ability and children’s spatial skills (Zhang, 2013; Kang et al., 2020). It might be that building a higher construction structure required children to engage in more discussion and communication, which naturally increased the frequency of their spatial language. Therefore, adults can make a certain assessment of the building skills of young children, make a reasonable sectionalization according to the ability of block building, control the number of young children entering the building block area, ensure the optimal configuration of the block building skills of young children, make full use of the role of the community, and improve the relative probability of spatial language and peer influence among young children in the same group. Adults should also create rich building situations for young children, use goal-directed block play as a means of introducing and acting out spatial concepts and relationships (Ferrara et al., 2011), guide children’s building themes and skills, and encourage children to complete higher construction structure. Furthermore, adults should guide young children to perceive and describe changes in spatial graphics and structures, pay attention to the spatial environment, and strengthen their spatial concept and experience.

Another finding was those young Chinese children who made cooperative forms used spatial language more frequently. A possible reason was that cooperative construction could lead to more peer interaction and prompt young children to share and negotiate building structure and solution strategies, such as how to obtain building blocks of various shapes, how to maintain the balance and symmetry of buildings, how to represent things in real life and other issues, all of which involve interactions of spatial language.

A cooperative and pleasant play atmosphere could encourage young children to use more spatial language for communication, stimulate spatial language among peers. Building together provided young children with opportunities to communicate, listen and discuss with each other. The children began to accept group rules, divide work and cooperate. Even if there were differences, they would try to solve them through consultation. Naturally, young children learned to share, respect others, and develop altruistic behavior. This provided an excellent context to cultivate their concentration and help them experience division and cooperation (Hu, 2018). Peers who are experienced in social interaction can also develop the construction and communication skills of children (Sluss and Stremmel, 2004). Therefore, adults should pay attention to the role of peers, encourage cooperation among peers, teach young children the expressive skills of spatial language, and support the discussion of spatial language among children.

In the present research, the spatial language used by young Chinese children had a relationship with age and class. Spatial features or properties, deictic terms, spatial locations, and spatial orientations or transformations of children in the younger class were significantly lower than those in the older class. This indicates that attention should be paid to the development of spatial language among younger and middle-class children. Previous studies had shown that biological maturity played an important role in the development of young children’s spatial concepts in early childhood (Zhao, 2007), such as the self-centered spatial coding ability at 3–5 years of young Chinese children increased significantly with age and made a significant leap from 4 to 5 years (Wang, 2009). Those aged 4–5 years old also had a rapid development period in the ability to recognize low level of spatial shapes (Li et al., 1997). Moreover, multiple studies had shown the relationships between spatial skills and spatial language at 4 years of age (Dessalegn and Landau, 2008, 2013). Studies have demonstrated that 4–5 years is a sensitive period for young children’s spatial ability development (Li et al., 1997; Wu et al., 2019), and a critical period for young children’s spatial language development. For a long time, collective teaching was an important form of educational organization in Chinese kindergartens (Qi, 2009; Zhu, 2011, p. 54). Accordingly, adults should pay attention to the class environment in younger age and middle-class settings, and the performance and features of the spatial language of young children’s block play. Meanwhile, adults should attach importance to children’s learning according to the children’s age, experience level, interests and needs (Yang, 2020), to create an appropriately spatial environment.

The present study found that the representation of shapes showed marginally significant differences in children in terms of gender. Young children’s perception of the different shapes of blocks was the embodiment of their application value and regularity in real life, and young children had different ways of using and representing blocks of different shapes. For instance, children of different genders used different symbols to represent blocks of the same shape: girls used “V”-shaped blocks to represent “flowers and grass,” and boys used them to represent “Mazda” (a car symbol). The potential reason was that the accumulation of gender differential spatial experience for male and female subjects (Chan, 2007). This might be related to young children’s gender roles and daily life experiences. The different requirements of social gender roles affected young children’s interests in different things (Hu, 2018) and their different representations of the same shape.

## Limitations and Future Research

This study conducted cross-sectional research of young children’s spatial language in block play. In the future, a longitudinal study of young children’s spatial language should be conducted to examine the impact of the abilities and forms of block building on spatial language and analyze the relationship between peer communication and the production and development of spatial language. Moreover, future research should expand the selection range to sample sizes of different construction structures and forms of block building and increase the number of participants so that the research results are more representative. Although the participants were all from the same type of kindergarten, their family educational environment, parenting style, and family economic level differed. Therefore, the variables of the family educational environment, parenting patterns, temperament types, and family economic level should also be examined. Future studies should analyze other variables synthetically to make the experimental results more rigorous.

## Data Availability Statement

The raw data supporting the conclusions of this article will be made available by the authors, without undue reservation.

## Ethics Statement

The studies involving human participants were reviewed and approved by Office of Scientific Research, Faculty of Education at Beijing Normal University and Kindergartens involved of China.

## Author Contributions

XY and YP designed the research and collected data for analysis. XY analyzed the data. YP provided crucial guidance. All authors were involved in interpretation and provided critical feedback and helped shape the research, analysis, and manuscript. All authors drafted the work and approved the published version.

## Conflict of Interest

The authors declare that the research was conducted in the absence of any commercial or financial relationships that could be construed as a potential conflict of interest.
